# Local Gamma Activity During Non-REM Sleep in the Context of Sensory Evoked K-Complexes

**DOI:** 10.3389/fnins.2019.01094

**Published:** 2019-10-15

**Authors:** Marco Laurino, Andrea Piarulli, Danilo Menicucci, Angelo Gemignani

**Affiliations:** ^1^Institute of Clinical Physiology, National Research Council, Pisa, Italy; ^2^Department of Surgical, Medical, Molecular Pathology and Critical Care Medicine, University of Pisa, Pisa, Italy; ^3^Coma Science Group, GIGA Consciousness, University of Liège, Liège, Belgium

**Keywords:** K-complex, slow oscillation, sensory processing, consciousness, source localization, gamma activity, sleep, down state

## Abstract

K-complexes (KCs) and Sleep Slow Oscillations (SSOs) are the EEG expression of neuronal bistability during deeper stages Non-REM sleep. They are characterized by a deep negative deflection lasting about half-a-second, sustained, at the cortical level, by a widespread and synchronized neuronal hyperpolarization (i.e., electrical silence). The phase of hyperpolarization is followed by a period of intense and synchronized neuronal firing (i.e., depolarization phase) resulting at the EEG level, in a large positive deflection (lasting about 0.5 s) and a concurrent high frequency activity (i.e., spindles). Both KCs and SSOs rather than being “local” phenomena, propagate over large sections of the cortex. These features suggest that bistability is a large-scale network phenomenon, possibly driven by a propagating excitatory activity and involving wide populations of synchronized neurons. We have recently shown that KCs and SSOs include a positive bump preceding the negative peak and that for sensory-evoked KCs this bump coincides with the P200 wave. We demonstrated that the P200 has a sensory-modality specific localization, as it is firstly elicited in the primary sensory areas related to the stimulus, which in turn receive projections from the thalamic core. We observed that the P200 acts as a propagating excitatory activity and hypothesized that it could play a key role in inducing the opening of K+ channels, and hence the cortical hyperpolarization. Here we demonstrate that the P200 is sustained by a high-frequency excitation bringing further support to its role in triggering bistability. We show that the P200 has a higher power density in gamma band as compared to the P900 coherently for all sensory modalities, and we confirm that the latter wave is crowned by higher activity in sigma-beta bands. Finally, we characterize the P200 gamma activity at the cortical level in terms of spatial localization and temporal dynamics, demonstrating that it emerges in sensory stimulus-specific primary areas and travels over the cortical mantle spreading toward fronto-central associative areas and fading concurrently with the N550 onset.

## Introduction

Bistability is the activity modality proper of thalamic and cortical neurons during Non-REM (NREM) sleep (Vyazovskiy et al., 2007). This dynamic pattern consists of an alternation between states of neuronal hyperpolarization-electrical silence (down states) and states of depolarization characterized by intense neuronal firing and synaptic activity (up states) (Steriade et al., 1993; Compte et al., 2003; Steriade and Timofeev, 2003). In humans, the down state lasts a few hundred milliseconds, and is followed by an up state of comparable duration. This bistable phenomenon is the neurobiological mechanism underlying both K-complexes (KCs) and Sleep Slow Oscillations (SSOs) (Amzica and Steriade, 1997a).

KCs and SSOs have been identified as synchronized-cortical-network phenomena (Amzica and Steriade, 1997b), involving large assemblies of neural circuits, so that their electrical patterns are easily detectable using surface EEG. Each KC/SSO is composed by a sharp negative wave (down state) followed by a large positive deflection crowned by high frequency activity (up state). While the former pattern is the EEG manifestation of a single event of bistable activity, the SSOs can either occur as single events or as trains of waves during periods of deepest NREM sleep (Crunelli and Hughes, 2010).

Several studies (e.g., Sejnowski and Destexhe, 2000; Steriade, 2004; Destexhe et al., 2007; Menicucci et al., 2013) have highlighted similarities between up states and wakefulness as they are both characterized by high-frequency activity with a similar spatio-temporal synchronization. In this framework, the up state high frequency activity has been hypothesized to play a fundamental role in memory consolidation (Mölle et al., 2011), with mentation as byproduct (Steriade, 2000).

The SSO/KC seem thus to subserve two different yet complimentary functions as it (1) maintains sleep and unconsciousness (down state), (2) plays a key role in the consolidation of recently acquired learning (up state).

While SSO/KC down and up states have been widely investigated unveiling the neuronal and network phenomena subtending their appearance and identifying their respective functions, the electrophysiological mechanisms responsible for the down state's ignition still remain to be fully elucidated.

In a previous study focused on temporally isolated SSOs events (Menicucci et al., 2013), we demonstrated that the down state is preceded by an early positive wave, that we named pre-down state. We showed that this early wave is characterized by the presence of a concurrent excitatory activation (beta to gamma) and hypothesized that this high-frequency excitatory activity could have a key role in triggering the opening of activity-dependent K+ channels, hence promoting the down state onset (Fröhlich et al., 2006; Sanchez-Vives et al., 2010).

During NREM sleep, simple sensory stimuli can evoke KCs, whose electrophysiological pattern consists of three distinctive waves: P200, N550, and P900 where the letter P (N) stands for positive (negative) deflection, and the number identifies the conventional latency of the wave peak in milliseconds (see Laurino et al., 2014).

The KC was first described by Loomis and colleagues (Loomis et al., 1935), and deeply investigated by Halász in recent years (Halász, 2005, 2016).

Amzica and Steriade (1997b), demonstrated that KCs and spontaneous SSOs originate from the same neurobiological mechanisms (hence the name evoked-SSOs for KCs), the KC counterparts of the down and up states corresponding, respectively to its N550 and P900 components (Laurino et al., 2014).

In Laurino et al. (2014), by using high-density EEG, we studied the quenching of sensory processing during NREM sleep in humans. We considered both stimulation events characterized by the evoked KC (eKC), elicitation (i.e., down and up state phases) and those where no response was evoked. We showed that, in both cases, the P200 is detectable and has the shortest latencies in stimulus-related primary sensory areas, acting as a wake-like activation traveling from primary sensory areas toward fronto-central regions. We observed that the elicitation of the biphasic component of the eKC (N550 and P900, respectively) depends on the P200 amplitude when it reaches areas with higher proneness to bistability (i.e., fronto-central areas), corresponding at the cortical level, to the insula and medial cingulate cortex Murphy et al., 2009. On this basis, we hypothesized that the triggering of the electrical silence (N550/down state) depends on the interaction between the level of excitatory activity sustaining the P200 (the higher the level, the higher the probability of triggering a down state) and the local proneness to bistability.

In this study, using the same eKC model of Laurino et al. (2014), we demonstrate that the P200 is a bottom-up excitatory cortical response to a sensory stimulation, while the P900 is associated to recursive cortico-thalamo-cortical activities. To this aim, we characterize and compare the P200 and P900 waves considering their spectral content at high frequencies (from sigma, to gamma bands) both in primary sensory areas and in fronto-central regions. We show that the P200 is characterized by higher gamma activity as compared to the P900, consistently across sensory modalities, and that this activity is already present in primary sensory areas and maintained throughout its cortical travel toward fronto-central regions. Conversely, we find that the P900 is characterized by higher sigma-beta activity as compared to the P200, consistently across sensory modalities and regions of interest. We finally characterize the P200 gamma activity at the cortical level in terms of temporo-spatial dynamics, demonstrating that it originates in stimulus-related primary sensory areas and travels over the cortex reaching fronto-central regions. These findings taken together suggest that the P200 is a purely cortical response composed by a slow positive deflection complemented by a concurrent excitatory activity in gamma band. We hypothesize that this excitatory activity acts as a trigger for the opening of K+ channels, thus favoring the local fall in down state and hence the quenching of sensory processing.

## Methods

### Participants and Experimental Protocol

This work aims at shedding light on aspects of evoked K-Complexes not investigated in Laurino et al. (2014), where the focus was set on latencies/topologies of P200, N550, and P900. We first briefly recall methods and experimental protocols developed in our previous research that are used also in this study (for a detailed characterization of subjects and stimulation modalities, please refer to Laurino et al., 2014).

Fourteen healthy volunteers (right-handed males, age 20–26 years) without history of medical diseases, psychiatric or neurological disorders, audiological, and/or visual deficits were included in the study. Each subject slept for two consecutive nights in the sleep laboratory (the first as an adaptation night). High-density sleep EEG recordings were collected in the second night (experimental session). The Local Ethical Committee approved the study, and the experimental procedures were in line with the tenets of the declaration of Helsinki.

During each experimental session, we performed a real-time EEG visual scoring continuously assessing the sleep stage (AASM criteria, Iber, 2007). Stimulation was carried out during N2 and N3 stages, but not during N1, awakenings or arousals, and consisted in sequences of auditory, tactile and visual stimuli delivered in a randomized order, with inter-stimulus intervals randomly sampled from a uniform distribution of allowed time intervals ranging from 15 to 20 s.

Acoustic stimuli were pure tones at 1,000 Hz with a 50 ms time-duration (rise and fall times 5 ms), delivered through in-ear headphones (XBA, Sony) and a sound pressure intensity of 60 dB. Tactile stimuli were achieved using mechanical vibrations (pallesthesia) at 300 Hz (50 ms of stimulation, rise and fall times 20 ms), delivered through an electrically driven vibrating device placed on the middle finger of the right hand. Visual stimuli consisted in light flashes (time-duration <1 ms) delivered with a photographic flash (32 Z-2, Metz), oriented toward the room ceiling.

### EEG Recordings

High-density EEGs were recorded using a Net Amps 300 system (GES300, Electrical Geodesic Inc., Eugene, OR, USA) with a 128-electrode HydroCel Geodesic Sensor net.

EEG signals were acquired with a sampling rate of 500 Hz in the 0.01–500 Hz band, using Net Station software, Version 4.4.2 (Electrical Geodesic Inc., Eugene, OR, USA), taking the vertex as the online reference potential (Cz). Electrodes impedance were maintained below 50 KΩ throughout the recording, in line with Electrical Geodesics recommendations.

All offline analyses were conducted using tailored Matlab codes (MathWorks, Natick, MA, USA), and when dealing with cortical sources reconstruction, taking advantage of Brainstorm toolbox functions (Tadel et al., 2011) and of OpenMEEG software (Kybic et al., 2005; Gramfort et al., 2011).

### Temporal Segmentation of eKCs

EEG sleep recordings were offline visually scored according to the AASM scoring criteria (Iber, 2007). After the removal of artifacted epochs, EEG signals were re-referenced to the mastoids' average, and trials consisting of full-fledged and temporally isolated eKCs (i.e., without any SSO event or delta wave occurring from 3 s before to 4 s after the eKC negative peak, see Laurino et al., 2014), were extracted.

**Figure 1:**
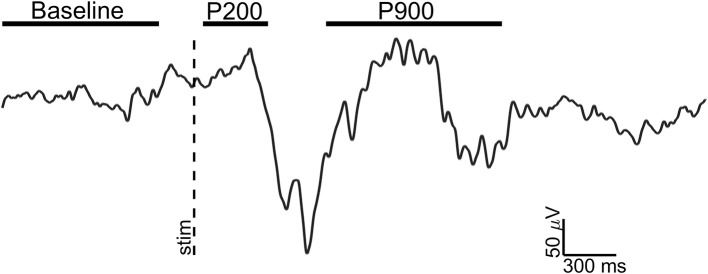
Exemplary EEG trace (Fz electrode, signal filtered in 0.3–30 Hz band) of a visual evoked K-complex for showing the time windows of analysis. The stimulus onset (vertical dotted line) and time windows considered for Baseline (1 s), P200 (300 ms), and P900 (1 s) are shown.

For each retained event, three epochs of interest were selected (see Figure 1):

1. Baseline: 1 s window ending 100 ms before the stimulus;
2. P200: 300 ms window starting 50 ms after the stimulus (pre-down state);
3. P900: 1 s window starting 750 ms after the stimulus (up state);

### Spectral Characterization of High Frequency Activity in Baseline, T200, and T900 Epochs

In line with the study hypotheses (the characterization of P200 and P900 waves considering their spectral content at high frequencies), three bands of interest were selected: sigma (9–18 Hz), beta (18–30 Hz), and gamma (30–45 Hz). Power spectra were estimated for each epoch of interest using the Fast Fourier Transform applied on a 300 ms Hamming-weighted sliding window. The length of the window was chosen as a trade-off between two opposite requirements: (i) provide an appropriate frequency resolution for a reliable power density estimation in the bands of interest, (ii) the window had to be wide enough to encompass the P200 wave and at the same time its time-limits had to be at a suitable distance both from the stimulus delivery and from the N550 peak.

For each subjects, electrode and epoch, band-limited power spectra were calculated as the sum of the power spectra over the frequency bins included in the band and then log-transformed.

For the P200, a single window was applied (the window length being equal to the epoch duration), while for the Baseline and the P900, power spectra were estimated using a 300 ms sliding window with a 33% overlap between contiguous ones. In this latter case (e.g., Baseline and P900), the mean power was obtained averaging among the windows pertaining to the epoch. The mean power densities in sigma, beta, and gamma bands were thus estimated for each subject, sensory-modality, electrode, and epoch (e.g., Baseline, P200, and P900), averaging over the related trials.

Two scalp regions of interest (ROI) were selected:

1. Primary Sensory Areas (PSA): electrodes over primary sensory areas with respect to each type of sensory stimulation: (i) electrodes related to acoustic stimuli were located over Brodmann areas (BAs) 41 and 42, (ii) electrodes related to tactile stimuli over BAs 3, 1, 2, and (iii) electrodes related to visual stimuli over BAs 17 and 18. Of note, these electrodes were those showing the shortest P200 latency in Laurino et al. (2014).
2. Frontal Central Areas (FCA): electrodes over fronto-central regions as these areas are characterized by a higher proneness to fall in down state (steeper Slope 1 in Laurino et al., 2014).

For each ROI, the mean power for each band of interest was estimated averaging among those of the electrodes included in the ROI.

### NREM Sleep Band-Wise Comparisons

For each sensory modality, band-wise differences between the three epochs (baseline, P200, and P900) were evaluated for both FCA and PSA sites.

Pairwise comparisons between all couples of epochs were conducted for each band using two-sided Wilcoxon signed rank tests. *P*-values significance threshold was set at 0.05.

For each band and ROI three comparisons were thus performed:

- P200 vs. Baseline.
- P900 vs. Baseline.
- P200 vs. P900.

### Cortical Source Localization of Gamma Activity

Based on the hypothesis of the P200 being a purely cortical wake-like activity, we focused the P200 cortical source analyses on gamma band.

EEG signals were first band-pass filtered (Hamming filter with zero-phase lag) in gamma band (30–45 Hz). Note that the choice of the time-windows length for cortical level analyses is not constrained by any trade-off between frequency and temporal resolutions, allowing thus a more precise windowing. For cortical level characterization, the P200 epoch was defined as the time interval from 140 to 350 ms after the stimulus: the P200 was divided in three time-windows based on the P200 template, obtained averaging across stimulus modalities (see **Figures 3A**, **4A**, **5A**): (i) T140 (+140 to +210 ms); (ii) T210 (+210 to +280 ms); (iii) T280 (+280 to +350 ms).

Standardized cortical current densities of gamma signals were estimated applying the standardized low-resolution brain electromagnetic tomography (sLORETA, Pascual-Marqui, 2002) algorithm, using Brainstorm toolbox for Matlab (Tadel et al., 2011). A default anatomical MRI template “Colin27” (Holmes et al., 1998) was used to estimate a symmetrical boundary element head model with 15002 dipoles (modeled layers: cortical surface, inner skull, outer skull, and scalp) using OpenMEEG software (Kybic et al., 2005; Gramfort et al., 2011).

For each trial, the time-course of the cortical standardized current densities was estimated using sLORETA algorithm. For each subject and sensory modality, the mean time course of standardized current densities was estimated averaging across the related trials. Z-scores of averaged cortical sources time-course of P200 were estimated with respect to the corresponding Baseline. As a last step the mean z-scores of the three time-windows (T140, T210, and T280) enclosing the P200 were estimated for each sensory modality and subject, averaging among the time-samples pertaining to the window (see **Figures 3B**, **4B**, **5B**). Voxels with z-scores > 1.65 (*p* < 0.05, one-tailed) were considered significant. For each sensory modality and time-window, only cortical areas composed of at least 100 adjacent significant voxels were retained, avoiding thus spurious findings. From here on, we will refer to gamma z-scores at the cortical level as gamma activations.

## Results

**Figure 2:**
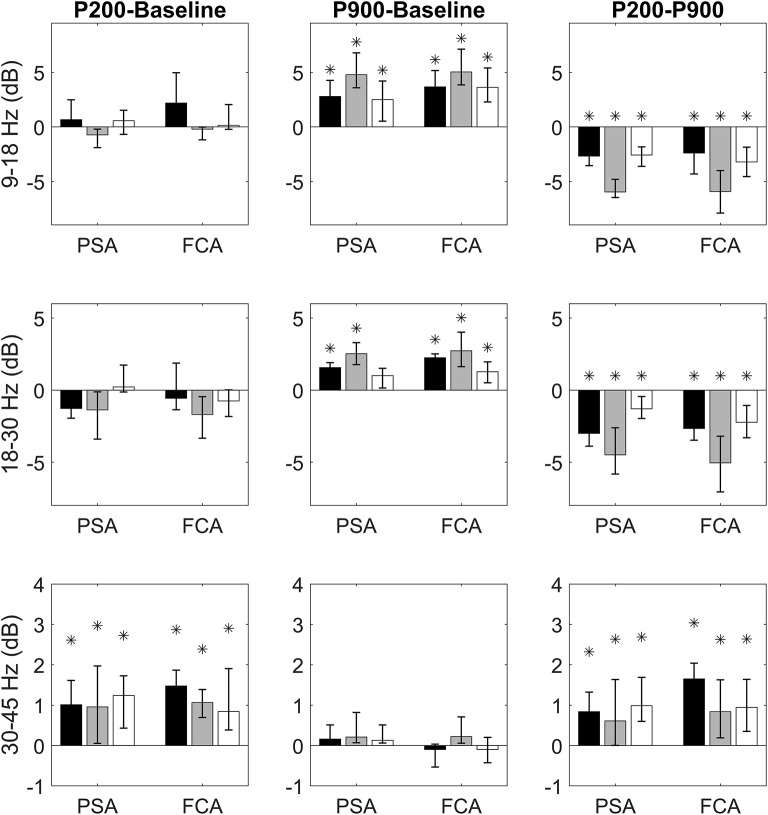
Bandwise comparisons between couples of epochs are presented for PSAs and FCAs. Each bar represent the band-wise difference of spectral powers between P200 and Baseline (first column), P900 and Baseline (second column), and P200 and Baseline (third column). The bar color identifies the sensory modality: acoustic (black), tactile (gray), and visual (white). Comparisons in sigma (9–18 Hz), beta (15–30 Hz), and gamma (30–45 Hz) bands are presented, respectively in the first, second, and third row. Each comparison is performed for two regions of interest: Primary Sensory Area (PSA) and Fronto-Central Area (FCA). The PSA bars are related to only the electrodes of specific sensory modality (black PSA bars for acoustic PSA, gray PSA bars for tactile PSA, and white PSA bars for visual PSA). Data are expressed as median values and interquartile ranges. Significant (pairwise Wilcoxon signed rank test, *p* < 0.05) comparisons are highlighted by asterisks.

We first verified whether the P200 and the P900 had different power at high frequencies with respect to the Baseline. For each band of interest (sigma, beta, and gamma), ROI (FCA and PSA) and sensory-modality (acoustic, tactile, visual), the power contents of the P200 and P900 were compared to that of the Baseline (Figure 2).

### The P200 Shows a Massive Enhancement of Gamma Power

Comparisons between P200 and Baseline epochs yield three main findings:

1. no difference in sigma and beta power is found either for PSAs or FCAs coherently for all sensory modalities;
2. a significant increase of gamma power with respect to Baseline is observed consistently across all sensory modalities. This increase is already apparent in PSAs (the site of P200 emergence) and is maintained in FCAs (Figure 2, first column).

### The P900 Is Accompanied by a Concurrent Increase of Power in Sigma-Beta Bands

Two main results emerge when considering P900-Baseline comparisons:

1. The P900 is characterized by a significant increase of power in sigma and beta bands. This increase holds for all sensory modalities both when considering PSAs and FCAs.
2. No significant difference between P900 and Baseline is apparent when considering gamma band (Figure 2, second column).

### Band-Specific Differences Between P200 and P900

Finally, we focused on the identification of spectral differences between P200 and P900. The P200 shows higher power within gamma band while P900 is characterized by significantly higher power in sigma-beta bands. Both findings are consistent across sensory-modalities and ROIs (Figure 2, third column).

### Source Localization of P200 Gamma Activity

**Figure 3:**
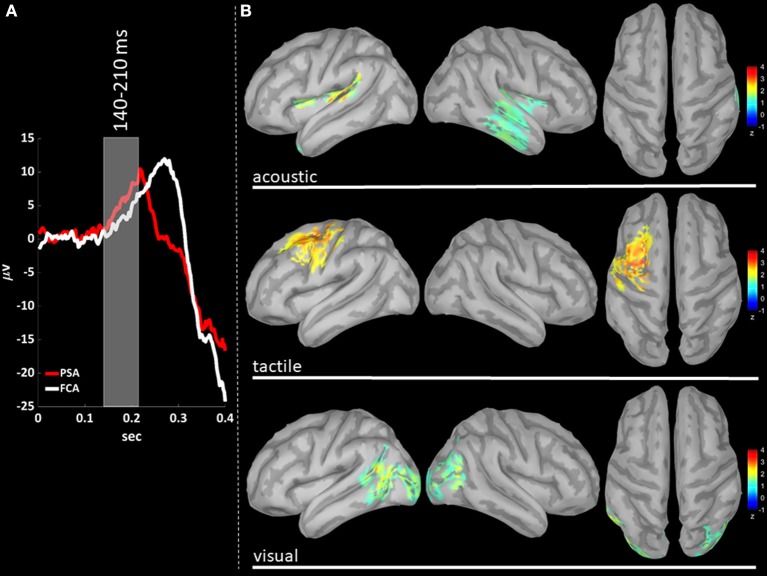
**(A)** The time-courses of grand mean eKCs templates (averaged over sensory modalities) from the stimulus delivery to 0.4 s after the stimulus are presented for Primary Sensory Areas (PSAs, red line) and for Fronto-Central Areas (FCA, white line). The T140 time window (+140 to +210 ms) is identified by a gray panel. **(B)** Standardized cortical current densities of gamma (30–45 Hz) signals evaluated over the T140 time-window for acoustic (first row), tactile (second row), and visual (third row) sensory modalities. The standardized current densities are expressed as the mean z-scores averaged over the T140 with respect to the corresponding Baseline (see Figure 1). For each sensory modality, only cortical areas composed of at least 100 adjacent significant voxels were retained.

**Figure 4:**
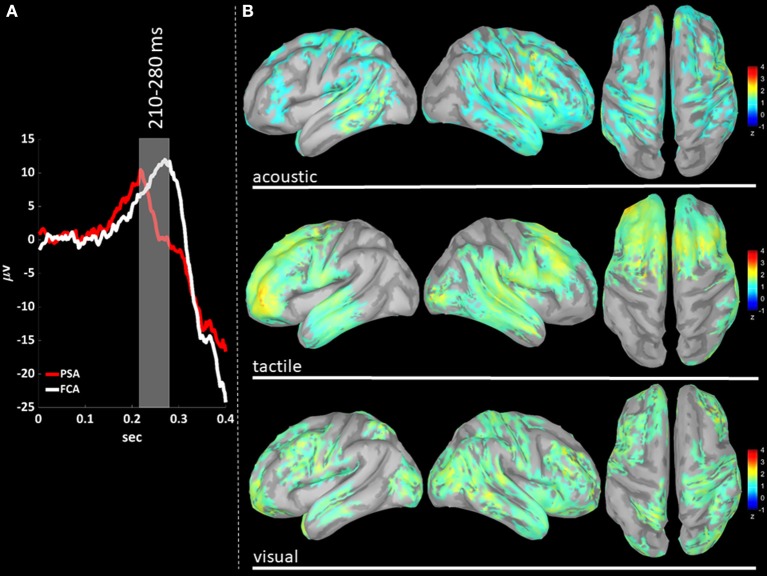
**(A)** The time-courses of grand mean eKCs templates (averaged over sensory modalities) from the stimulus delivery to 0.4 s after the stimulus are presented for Primary Sensory Areas (PSAs, red line) and for Fronto-Central Areas (FCA, white line). The T210 time window (+210 to +280 ms) is identified by a gray panel. **(B)** Standardized cortical current densities of gamma (30–45 Hz) signals evaluated over the T210 time-window for acoustic (first row), tactile (second row), and visual (third row) sensory modalities. The standardized current densities are expressed as the mean z-scores averaged over the T210 with respect to the corresponding Baseline (see Figure 1). For each sensory modality, only cortical areas composed of at least 100 adjacent significant voxels were retained.

**Figure 5:**
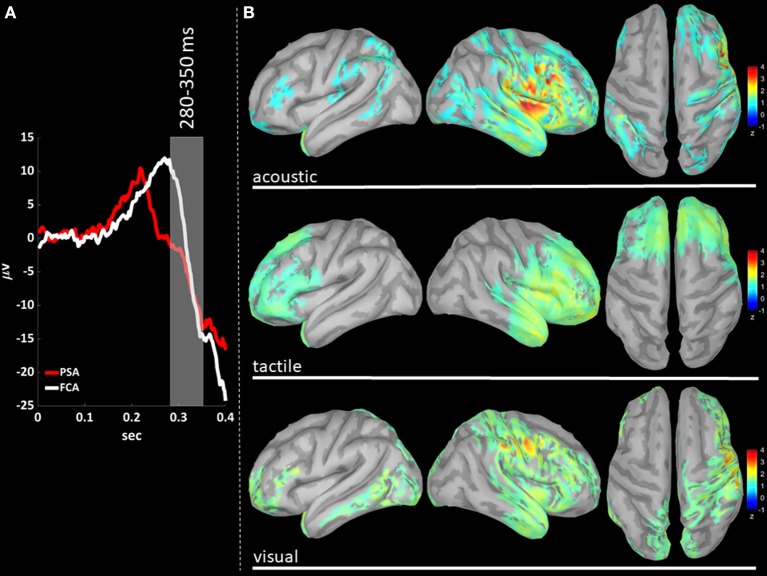
**(A)** The time-courses of grand mean eKCs templates (averaged over sensory modalities) from the stimulus delivery to 0.4 s after the stimulus are presented for Primary Sensory Areas (PSAs, red line) and for Fronto-Central Areas (FCA, white line). The T280 time window (+280 to +350 ms) is identified by a gray panel. **(B)** Standardized cortical current densities of gamma (30–45 Hz) signals evaluated over the T280 time-window for acoustic (first row), tactile (second row), and visual (third row) sensory modalities. The standardized current densities are expressed as the mean z-scores averaged over the T280 with respect to the corresponding Baseline (see Figure 1). For each sensory modality, only cortical areas composed of at least 100 adjacent significant voxels were retained.

We performed a cortical current source localization of gamma oscillations (30–45 Hz), for the three time-windows (T140, T210, T280) encompassing the P200 wave. Standardized current densities were transformed in z-scores (gamma activations) with respect to the Baseline epoch. In Figures 3A, 4A, 5A shows the grand-average templates of P200 for PSA (red) and FCA (in white) along with the analyzed time-window (T140 in Figure 3; T210 in Figure 4; T280 in Figure 5). Of note, the PSA's P200 peak precedes that of the FCA, indicating that PSA are always the first regions activated by thalamic excitatory sensory volleys. Figures 3B, 4B, 5B show for each sensory modality, cortical sources with significantly higher gamma activity as compared to Baseline. Figures 3B, 4B, 5B show the three consecutive phases of gamma cortical travel. In T140, significant gamma activations are predominantly localized over the stimulus-specific primary sensory areas (Figure 3B). When considering the interval spanning from 210 to 280 ms (T210, see Figure 4B), gamma activations spread from primary sensory areas toward central and anterior cortical regions coherently for all sensory modalities. Depending on the sensory modality, other cortical areas are involved:

- For the acoustic modality, further activations are observed in parieto-temporal areas including the secondary auditory cortex (superior edge of the lateral surface of the temporal lobe).
- In the tactile modality, the gamma activity spread involves also areas of the temporal gyri.
- In the case of visual modality, gamma activity spread includes large portions of the occipital lobe and of parieto-temporal regions.

In T280 time-window (Figure 5B), gamma activity is still detected mainly in fronto-central regions, even if an overall reduction of activations can be observed for all areas activated during the T210 time-window (this finding holds for all sensory modalities). Notably the T210, which includes the peaks of the P200 slow positive deflection, is the time-window where gamma activations spread over larger portions of the cortical mantle as compared to either T140 or T280.

## Discussion

Bistability is a paradigmatic dynamic of brain activity during NREM sleep, subtending both SSOs and KCs, which have been identified as highly synchronized-cortical-network phenomena involving large neuronal patches by Amzica and Steriade (1997b).

We have previously demonstrated that both SSOs (Menicucci et al., 2013), and KCs (Laurino et al., 2014) are triphasic waves, as the classical bistable pattern (down-state/N550, up-state/P900), is preceded by an early positive deflection (pre-down state/P200). It is fair to underline that the identification of the KC as a triphasic pattern (P200, N550, and P900) is not a novelty, as it was already observed by Loomis et al. (1935), and investigated by Roth et al. (1956). However, an exhaustive characterization of the P200 wave (and of its SSO counterpart, i.e., pre-down state) and the identification of its role, have been overlooked until recent years.

The role of P200, using the evoked K-complex model, was investigated by Riedner et al. (2011), and thoroughly characterized by our group (Laurino et al., 2014). We described temporal and morphological features of evoked KCs for three sensory modalities: acoustic, tactile and visual, demonstrating that the classical biphasic components of the KC (N550 and N900) are independent from the stimulus' sensory modality, showing a higher detection rate in fronto-central areas. On the contrary, we observed that P200 topology is highly dependent on the stimulus sensory modality, with the earliest waves detected in the related primary sensory areas. We further demonstrated that the P200 is a wave rapidly traveling as a cortical excitatory activity over the whole cortex. We hypothesized that the P200 acts as a traveling cortical excitation whose function is the triggering of the bistable cortical response (N550-P900) which is in turn critical for quenching sensory processing (maintaining thus sleep and unconsciousness, N550/down-state), and favoring the consolidation of newly acquired abilities (spindle activity concurrent with the up-state).

As elegantly summarized by Halász (2016), “KCs have a Janus-faced nature: on the one hand they are evoked by an input-dependent traveling cortical excitation and on the other hand they represent an induced, cortical slow wave in the fronto-central region.”

Several studies (e.g., Sejnowski and Destexhe, 2000; Destexhe et al., 2007) have characterized high-frequency activity crowning up states demonstrating its similarity to cortical activity during wakefulness. This high frequency activity (e.g., spindles), has been hypothesized to be deeply engaged in memory consolidation processes (Schabus et al., 2004; Steriade, 2006; Mölle et al., 2011; Lüthi, 2014).

On the contrary, the electrophysiological mechanisms responsible for the down state's ignition and the possible role of P200 (pre-down state in SSOs), remain to be fully elucidated. Based on our previous findings (Menicucci et al., 2013; Laurino et al., 2014), we herein provide further evidence that the P200 and specifically its high-frequency activity is a plausible candidate able to favor the down state ignition, and we demonstrate that the gamma band component of the P200 has striking analogies with that proper of wakefulness.

In this study, using the same eKC model of Laurino et al. (2014), we characterize the high-frequency spectral components of the two active states of eKCs, namely P200 and P900 waves (see Figure 1). We next identify the spatio-temporal dynamics of gamma activity cortical sources during the P200 wave.

Four main original results emerge from our study:

- The P200 is characterized by a significant increase of gamma activity in sensory-modality-primary sensory areas as well as in fronto-central regions, as compared to Baseline and P900 (Figure 2).
- The P900 is characterized by a significant increase of sigma-beta activity in sensory-modality-primary sensory areas as well as in fronto-central regions, as compared to Baseline and P200 (Figure 2).
- The distribution of gamma cortical activations is dependent on the sensory modality, with earliest activations detected in the stimulation-related primary sensory areas (Figure 3).
- P200-related gamma activity travels on the cortical mantle as an excitatory activity (Figures 4, 5), reaching its maximum cortical spreading in the time-window enclosing the P200 peak, and partially receding in the T280 windows (concurrently with the down state onset).

Based on these results, a global picture emerges: while on the one side P200 and P900 (pre-down state and up state in spontaneous SSOs, respectively) are both driven by the interplay between thalamus and cortex, each wave is subtended by different physiological mechanisms subserving different functional roles.

Let us now discuss the emerging picture in terms of an ideal bottom-up travel of a sensory input from periphery to cortical areas, for P200, and an ideal “top-down bottom-up cycle” between thalamus and cortex for P900. This allows us to pinpoint each finding in a unifying scenario and in an intuitive order.

### P200 Gamma Activity as a Wake-Like Sensory Cortical Excitation

As described in our previous work (Laurino et al., 2014), the P200 is the first wave of the eKC, with shortest latencies detected in the primary sensory areas of stimulation. This result demonstrates that also during NREM sleep, sensory stimuli follow wake-like sensory pathways, ascending to primary sensory areas from core thalamic nuclei (Velluti, 1997): the specific wiring configuration of sensory inputs is arranged to minimize the processing time in term of perception during wakefulness (Kandel et al., 2000). The efficiency of this wiring configuration makes it the predominant sensory pathway also during NREM sleep (Laurino et al., 2014).

The hypothesized excitatory nature of the P200 gains further strength from the identification of a concurrent spectral component in gamma band: P200 gamma power is significantly higher both as compared to Baseline and to P900. This latter finding assumes a fundamental importance when considering that the high-frequency activities crowning the up states (P900) have been described as ‘fragments of wakefulness’ (Destexhe et al., 2007). This statement along with the fact that gamma rhythm is deeply involved in cortical processing during wakefulness (Bosman et al., 2014), strongly suggests that the gamma band rhythm proper of the P200 wave, is a real wake-like activity.

However, a limitation of our study should be noted: we did not stimulate systematically the subjects during wakefulness in order to show an analogous excitatory response with respect to NREM sleep. However, several studies have previously characterized the spectral activity of sensory processing in wakefulness. These studies have shown how specific gamma-band activity is typically evoked in human EEG as response to sensory stimulations.

During wakefulness, gamma oscillations are nearly ubiquitous, as they are involved in a wide range of low (neural circuits) and high level (e.g., cognition, emotion, sensory processing) brain functions (see Bosman et al., 2014).

Gamma rhythms can be induced by sensory stimuli: sniffing-induced gamma rhythms were first described in the olfactory bulb and pyriform cortex of the hedgehog by Nobel laureate Adrian (1942), and induced gamma activity following sensory-specific stimulation has since been identified in human's visual (Adjamian et al., 2004; Hoogenboom et al., 2006), auditory (Pantev et al., 1991; Pantev, 1995), and somatosensory (Chen and Herrmann, 2001; Bauer et al., 2006) cortices. The increase in gamma activity has been demonstrated to be sensory modality-dependent: auditory stimuli induce larger activations in the temporal cortex, visual stimuli in primary visual cortex, and tactile stimuli in the somatosensory cortex (Jefferys et al., 1996; Whittington et al., 2011).

Our findings fit nicely with this scenario, as we find that gamma cortical current distribution is dependent on the sensory modality, with the earliest cortical activations detected in the related primary sensory area (T140, Figure 3B).

One of the most intriguing roles of gamma band is its capability to synchronize distributed neuronal patches thus promoting and regulating the flow of information between neuronal groups and contributing to brain computations. The flow of information is facilitated by gamma synchronization as it entrains the firing rate of the involved neurons at frequencies of 40–80 Hz, obtaining a precise spike synchronization among neighboring excitatory neurons. These sequences of synchronized spikes propagate to other neuronal groups, where the synchronization of the incoming spikes enhance the feedforward coincidence detection process, thereby favoring their efficacy in recruiting and entraining other neurons (Salinas and Sejnowski, 2001). Gamma synchronization has also a modulatory function as it affects the efficacy of the synaptic input to the group of synchronized neurons: when a group of neurons is synchronized in gamma band, its activity is strongly influenced by gamma-rhythmic inhibitory inputs (Hasenstaub et al., 2005). Excitatory inputs efficacy thus depends on their timing with respect to inhibitory activity (i.e., excitatory inputs are most effective when arriving out of phase with respect to the inhibitory barrier, Fries, 2009).

Again, an analogy with wakefulness can be drawn, as we find that gamma activity, after emerging in the stimulus-specific primary sensory areas, travels over the cortex mantle reaching fronto-central integrative regions. We can reasonably assume that the gamma activity cortical travel is sustained and driven by the same neurobiological mechanisms promoted by gamma synchronization during wakefulness.

### P900 Sigma/Beta Activities Emerge From Cortico-thalamo-cortical Loops' Activity

The P900 is characterized by a significantly higher sigma and beta band power as compared to Baseline and P200. This finding is in line with an amount of studies both in animal models and humans (see Steriade, 2006), demonstrating that up states (P900 waves) are crowned by high frequency activity in sigma (spindle activity) and beta band.

From an electrophysiological point of view, the P900 slow positive deflection reflects the size of the underlying synchronized neuronal pool with membrane potentials shifting toward depolarization levels, a mechanism initiating in cortical neurons and reinforced cortico-thalamo-cortical loops (Timofeev and Chauvette, 2011). During the P900 (up state), both cortical mechanisms and volleys from subcortical structures (thalamus and hippocampus) facilitate the entry of Ca++ in dendrites of cortical neurons, thus favoring cellular and synaptic plasticity, which are the molecular mechanisms promoting the consolidation of newly acquired abilities (see Rasch and Born, 2013). Ca++ entry causes the depolarization of membrane potentials inducing a synchronized neural spiking, detectable by surface EEG as a high-frequency EEG activity crowning the up state mainly in sigma band (in the frequency range of thalamic spindle activity, see Lüthi, 2014), but extending also to beta (Mölle et al., 2002).

### P200 Gamma Activity as a Trigger for Bistability

We have previously hypothesized that the fast traveling P200 wave acts as a trigger for the bistable complex (N550/N900 of eKCs). We showed that P200 along its travel has a waxing behavior, entraining progressively larger neuronal patches (by synchronizing their activity), and displaying larger amplitude in fronto-central areas, as their integrative nature promotes an electrical reverberation of recently generated up-scaled Hebbian circuits (Tononi and Cirelli, 2006).

We here demonstrate that the P200 wave is complemented by a significant gamma activity spreading over the cortical mantle as an excitatory activity (see Figures 3–5). This finding suggests a plausible mechanism able to induce the following hyperpolarization phase (N550), thus reinforcing the hypothesis on the P200 complex as a traveling trigger for bistability (Laurino et al., 2014).

In the last two decades, a growing number of studies has dealt with the identification of the electrophysiological mechanisms responsible for the onset of electrically silent states (down-state/N550) and ultimately for preserving unconsciousness. Sanchez-Vives et al. (2010), demonstrated that cortical down states are promoted by the synchronous opening of activity-dependent K+ channels, and that both the speed of transition to the silent state and its duration are modulated by the preceding electrical activity.

In a previous study, Vyazovskiy et al. (2009), were able to induce SSOs by delivering intracortical electrical pulses in sleeping rats. They concluded that volleys of electrical activity capable of excite and recruit sufficiently large neural populations, induce SSOs (and specifically down states) during natural sleep in rats.

Lemieux et al. (2015) identified, in cats under ketamine-xylazine anesthesia, three mechanisms that promote the down state onset: (1) a reduction of excitatory synaptic activity in large neuronal pools with a nearly simultaneous decrease of cortical neurons' firing, (2) an increase of inhibitory activity in subpopulations of neurons; (3) the delivery of an excitatory input effectively triggering the transition. In their study, Lamieux and colleagues, mimicked excitatory inputs by delivering pulse currents to neocortical sites during active states, and were able to induce down states within tens of milliseconds from the stimulus onset.

Our hypothesis that P200 gamma activity spreading over the cortical mantle as a wake-like excitatory activity acts as a trigger for the onset of the down state is strongly supported by the above-mentioned findings. We conclude that also in humans' natural sleep, a sufficiently strong cortical excitatory electrical activity (able thus to excite and recruit large cortical neuronal populations) acts as a trigger for the down state ignition, hence preserving unconsciousness.

## Concluding Remarks

Here, using the sensory-evoked K-complex model, we provide strong evidence that an excitatory cortical activity concurrent with the P200 wave is able to trigger the down state ignition during natural sleep in humans. We show that the P200 is complemented by a concurrent cortical activation in gamma band acting as a wake-like excitatory activity emerging from sensory stimulus-related primary sensory areas, rapidly spreading over the cortical mantle reaching fronto-central associative regions and fading concurrently with the down state onset. These findings demonstrate that pathways and neurobiological mechanisms sustaining the P200-related gamma activity are completely different from those subtending high-frequency activities crowning up-states (e.g., cortico-thalamo-cortical loops). We show that the P200 has a significantly higher gamma power as compared to P900, and conversely the latter is characterized by significantly higher power in sigma-beta bands.

Our hypothesis of the role of excitatory gamma activity as a trigger for down states gains further support from previous studies in the animal model showing that volleys of electrical activity able of exciting and thus recruiting sufficiently large neuronal pools, induce down states.

In our opinion, the findings and the hypothesis herein presented assume a special interest as, differently from the above-mentioned studies, they are drawn from an investigation on naturally sleeping humans, thus in a more ecological context.

At this point, we believe some considerations are due. The finding about a wake-like behavior of sensory-stimulus induced gamma activity, in terms of both cortical origins and mechanisms of cortical spreading, in no way implies a conscious information processing.

While a generalization of the results we obtained using the sensory-evoked KC model to all SSOs seems appropriate, as they are subtended by very same neurobiological mechanisms (Amzica and Steriade, 1997a,b), future studies focusing on down state ignition in spontaneous sleep slow oscillations are warranted. In this framework, a previous study from our group (Menicucci et al., 2013), suggests that the down state ignition in spontaneous SSO could be triggered by mechanisms similar (e.g., excitatory activity), to those herein presented.

Finally, we would like to point out that, while the excitatory activity mechanism is effective in initiating the down state, results from studies in the animal model (see Lemieux et al., 2015), show that other concurring or even alternative neurobiological mechanisms and/or conditions can favor the down state onset.

## Data Availability Statement

The datasets generated for this study are available on request to the corresponding author.

## Ethics Statement

The studies involving human participants were reviewed and approved by Pisa local ethical committee. The patients/participants provided their written informed consent to participate in this study.

## Author Contributions

ML, AP, DM, and AG contributed to the design, implementation of the study, analysis of the results, and writing of the manuscript.

### Conflict of Interest

The authors declare that the research was conducted in the absence of any commercial or financial relationships that could be construed as a potential conflict of interest.
